# Hydrogen bonding capabilities of group 14 homologues of HCN and HNC†

**DOI:** 10.1039/c9ra00856j

**Published:** 2019-02-18

**Authors:** Joanatan M. Bautista-Renedo, Horacio Reyes-Pérez, Erick Cuevas-Yáñez, Carlos Barrera-Díaz, Nelly González-Rivas, Joel Ireta

**Affiliations:** Centro Conjunto de Investigación en Química Sustentable UAEM-UNAM km 14.5 Carrera Toluca-Atlacomulco, Campus UAEmex “El Rosedal” San Cayetano-Toluca 50200 Toluca de Lerdo Estado de México Mexico nelymagr@gmail.com; Departamento de Química, División de Ciencias Básicas e Ingenierías, Universidad Autónoma Metropolitana-Iztapalapa A.P. 55-534 México D.F. 09340 Mexico; División de Ingeniería Química, Tecnológico de Estudios Superiores de Jocotitlán Carretera Toluca-Atlacomulco km 44.8, Ejido de San Juan y San Agustín Jocotitlán Edo. México Mexico

## Abstract

This study is directed towards assessing hydrogen bond acceptor/donor capabilities of heavier group 14 homologues of HCN and HNC. A structural, energetic and topological study using *ab initio* (MP2, CCSD(T)), electrostatic potential (EP) and quantum theory of atoms in molecules (QTAIM) methodologies was carried out on HNX⋯HNX and HXN⋯HXN dimers and their respective monomers, where X = C, Si, Ge, Sn and Pb. The obtained results suggest the presence of weak hydrogen bonds in both kinds of complexes, and remarkably Ge and Sn act as unconventional hydrogen donors.

## Abstract 2

The hydrogen bond capabilities of heavier group 14 homologues of HCN and HNC are studied by means of (MP2 and CCSD(T)) methods. It is shown that homologues with donor atoms less electronegative than H are able to form hydrogen bonds.

## Graphical abstract

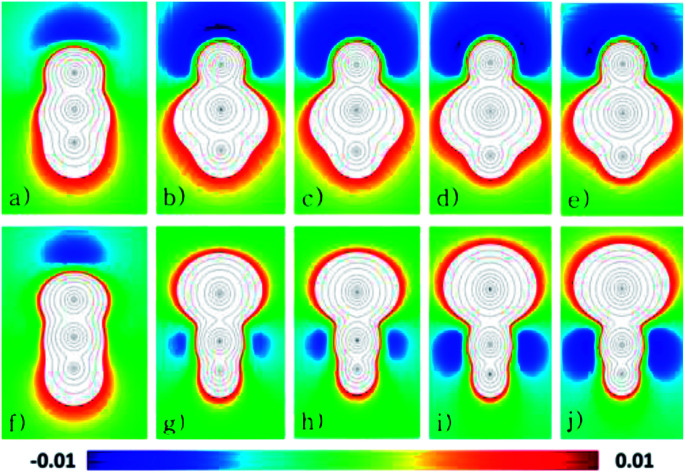

## 1. Introduction

Hydrogen bonds (HBs) are non-covalent interactions responsible for the peculiar physicochemical properties of diverse materials such as water and proteins.^1,2^ D–H⋯A, where D is the donor atom and A stands for the acceptor atom or region, is the customary representation of an HB. There has been a long-standing interest in determining the factors driving HB formation.^3^

The HBs formed by the usual D and A atoms are termed conventional. Nowadays the occurrence of HBs involving unconventional donor and acceptor atoms or groups has been well established with both theoretical and experimental studies.^4,5^ Four cases of unconventional HBs have been proposed: (a) HBs with unconventional donors such as C–H from both aromatic and aliphatic groups; (b) HBs with unconventional acceptors such as π-clouds or metals; (c) HBs with both unconventional donor and acceptor groups; and (d) dihydrogen bonds D–H⋯H–A.^6–8^ Unconventional HBs are largely recognized as important forces in the area of crystal engineering,^9^ protein structure and function^10^ or as relevant weak non-covalent interactions in anion recognition by discrete pre-organized receptors.^11^

**Fig. 1:**
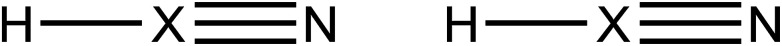
Homologues of HCN and HNC. Where X = C, Si, Ge, Sn and Pb.

Either in conventional or unconventional HBs it is considered the D atom to be more electronegative than H, as stated in the IUPAC definition.^2^ In this work we aim for establishing if a given D–H fragment must fulfil the latter in order to form a HB. Below it is shown that fragments in which the D atom is less electronegative than H form HBs. For that purpose it is investigated the hydrogen bonding capabilities of the heavier group 14 homologues of HCN and HNC (Fig. 1) using the second order Møller–Plesset perturbation theory (MP2), coupled-clusters with single and double and perturbative triple excitations (CCSD(T)). Based on an analysis of dimer association energies, its bond distances and bond directionality, the changes in frequencies and dipole moments upon dimer formation, and the values of the dimer electron density at bond critical points, it is argued that the interaction NXH⋯NXH, where X stand for C, Si, Ge, Sn and Pb, can be considered as a HB even though Si, Ge, Sn and Pb are less electronegative than H, whereas the interaction XNH⋯XNH can be considered as a HB only when X = C. Furthermore it is found that inspecting the electrostatic potential of NXH and XNH can be anticipated which molecules will form a HB, and in which case a proton transfer associated to the HB formation will be favoured. Thus, these results reveal a novel case of unconventional HBs and further our understanding of the nature of the HB interactions.

## 2. Computational details

The optimized geometry of monomers and interacting dimers is obtained using MP2 together with the correlation consistent basis set aug-cc-pVDZ (see ESI†). The absence of imaginary frequencies is corroborated for all the obtained geometries. Then these structures are used for assessing the extrapolated to the complete basis set (CBS) limit total energies at the MP2 and at the CCSD(T) level of theory, using the series of consistent basis sets aug-cc-pVnZ, where n = D, T and Q. The CCSD(T) calculations are carried out within the freeze core approximation. For calculating the CBS total energies (YCBS) it is used the next expression:^12^*Y*_(*x*)_ = *Y*_CBS_ + *A* e^−*ax*^where *x* = 2, 3, 4 for the double-zeta (D), triple-zeta (T) and quadruple zeta (Q) basis sets, respectively. *A* and *a* are fitting parameters and *Y*_(*x*)_ is the total energy of dimers or monomers obtained using the dimer basis set. Interaction energies are calculated using the CBS total energies as well as using the single-point total energies corrected due to the basis set superposition error (BSSE), which is calculated using the counterpoise correction. The dependence of the interaction energy with respect to the angle of the interaction is carried out at the CCSD(T)/aug-cc-pVDZ level of theory and considering the BSSE correction. All these calculations are carried out with the software NWChem 6.5.^13^ To characterize the interaction in the investigated systems, the electronic density of the dimers is analyzed following the quantum theory of atoms in molecules (QTAIM)^14^ and using the MultiWFN package.^15^

## 3. Results and discussion

Association energies of the investigated dimers are listed in Table 1. First we notice that independently of the level of theory used for calculating the interaction energies, it is clear that the formation of the HXN⋯HXN dimers (except for the HPbN⋯HPbN dimer) and the HNC⋯HNC one is energetically favorable, at least at 0 K.

**Table 1:** Association energies in kcal mol^−1^^a^

Dimer	Level of theory	DZ	TZ	QZ	CBS
(HCN)_2_	MP2	−4.41	−4.73	−4.84	−4.88
	CCSD(T)	−4.18	−4.59	−4.72	−4.68
(HSiN)_2_	MP2	−2.93	−3.51	−3.76	−3.68
	CCSD(T)	−4.47	−5.24	−5.50	−4.97
(HGeN)_2_	MP2	−2.63	−3.20	−3.46	−3.58
	CCSD(T)	−3.95	−4.74	−5.00	−4.91
(HSnN)_2_	MP2	−3.27	−3.81	−3.77	−3.49
	CCSD(T)	−2.85	−3.39	−3.34	−2.67
(HNC)_2_	MP2	−7.16	−7.68	−7.81	−7.93
	CCSD(T)	−6.26	−6.91	−7.04	−7.11
(HNSi)_2_	MP2	−0.20	−0.51	−0.58	−0.47
	CCSD(T)	−0.16	−0.50	−0.56	−0.42
(HNGe)_2_	MP2	0.22	−0.30	−0.47	−0.31
	CCSD(T)	0.26	−0.25	−0.38	−0.15
(HNSn)_2_	MP2	−0.03	−0.54	−0.76	0.12
	CCSD(T)	−0.26	−0.71	−0.85	0.07
(HNPb)_2_	MP2	0.14	−0.34	−0.46	10.96
	CCSD(T)	−0.51	−0.80	−0.82	4.14

a The first three columns correspond to basis set corrected association energies. In the last column are given association energies calculated using the complete basis set limit-extrapolated energies.

For the rest of the dimers the absolute value of the interaction energies is either less than 1 kcal mol^−1^ or even positive; *i.e.* these dimers may not be formed even at 0 K. The formation of the HPbN⋯HPbN dimer is not observed because it transforms to HNPb⋯HNPb owing to a proton transfer from Pb to N. Likely this proton transfer process is assisted by the formation of an Hb as it is discussed below. We also notice that the BSSE corrected interaction energies are quite similar to these calculated with the CBS total energies, except for the systems containing the two heaviest atoms here investigated, Sn and Pb. Excluding the systems containing the latter two atoms differences between the BSSE corrected and the CBS interaction energies are less than 1 kcal mol^−1^. The large differences between the BSSE corrected and the CBS interaction energies, for the systems containing Pb and Sn, are likely due to the slow convergence of the total energy with respect to the basis sets. For these system it will be desirable to calculate total energies with a quintuple zeta basis sets to be completely confident of the results obtained using the extrapolated energies to the CBS limit, nevertheless that is out of the computational possibilities of our group nowadays. Despite the agreement between the MP2 and the CCSD(T) interaction energies mentioned above, the dimer stability ordering is predicted differently by these two methods. In the case of the MP2 results the stability ordering is even dependent of the basis sets used. CCSD(T) predicts the same stability ordering of the energetically favorable dimers, independently of the basis set used. Thus according to the CCSD(T) results the stability ordering is HNC⋯HNC > HSiN⋯HSiN > HGeN⋯HGeN > HCN⋯HCN > HSnN⋯HSnN. In these set of dimers the largest interaction energy is predicted to be −7.11 kcal mol^−1^ for HNC⋯HNC and the lowest −2.67 kcal mol^−1^ for HSnN⋯HSnN at the CCSD(T)/CBS level of theory. Whether these interactions are HBs or not is scrutinized next.

**Fig. 2:**
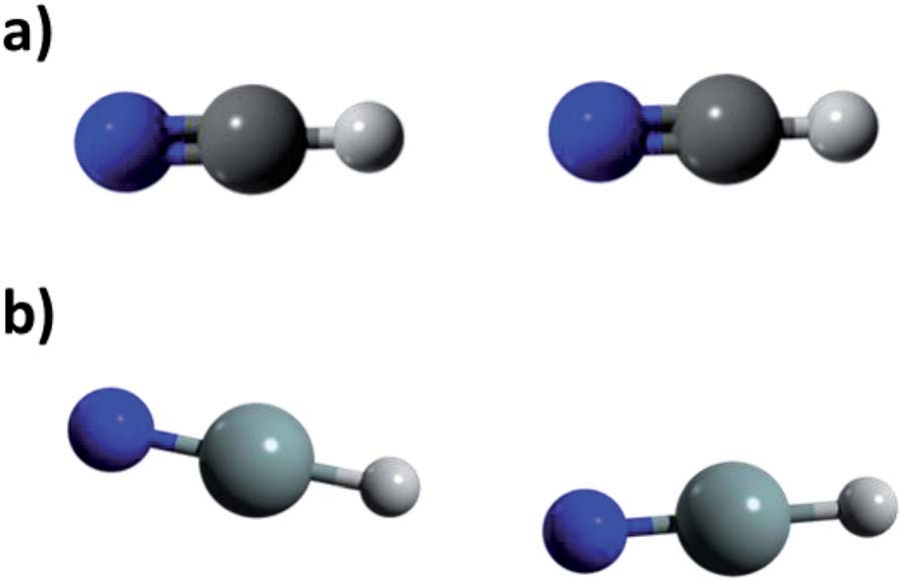
Arrangements found in calculated dimers through MP2/aug-cc-pVDZ methodology for HXN and HNX. (a) Lineal (HCN, HGeN and HNX) and (b) angular (HSiN y HGeN).

To determine if such interactions can be considered as HBs first we analyze the dimer optimized geometries. In Table 2 are listed the N⋯H and X⋯H distances, labeled as *d*_HB_, the angles the angles N⋯H–X and X⋯H–N, labeled as *ϕ*, and the angles X–N⋯H and N–X⋯H, labeled as *θ*. It is also listed the bond distances X–H and N–H in the dimers, labeled *b*, and by how much it differs for that distance in the monomer, labeled Δ*b*. Positive values of Δ*b* indicate that *b* is larger in the dimer than in the monomer. For the set of energetically favorable dimers all the *d*_HB_ values are smaller than 2.5 Å, which is considered in some works as the upper *d*_HB_ value in conventional hydrogen bonded systems (see *e.g.*ref. 16). For the dimers not included in the set of the energetically favorable ones the *d*_HB_ values are larger than 2.5 Å. Still considering the van der Waals radii given in ref. 17 it is found that the *d*_HB_ value in all the dimers is smaller than the sum of the van der Waals radii of H atom and the acceptor one, a criterion commonly used to identify the formation of HBs. The angle *ϕ* that accounts for the position of the acceptor atom with respect to the donor group is 180° in all the cases, which is the optimal value in HB bonded systems. The angle *θ* that accounts for the position of the proton with respect to the acceptor group is also 180° for all the cases except for the HSiN⋯HSiN and HSnN⋯HSnN dimers (see Fig. 2). The deviation from 180° in these two cases may be due to lateral electrostatic interactions as it is discussed below. Still these deviations in the *θ* angle should not affect the HB formation. Changes in *b* upon the dimer formation are usually found in HB bonded systems. The elongation of *b* range between 0.007–0.034 Å in the set of energetically favorable dimers, while for the rest of the dimers the change is around 0.001 Å, at most, either positive or negative or even it does not change at all. Considering that the change of ∼0.001 Å is negligible, these results suggest that the set of energetically favorable dimers is HB bonded.

**Table 2:** Geometric parameters commonly used for characterizing hydrogen bonds^a^

Dimer	*d*_HB_	*ϕ*	*θ*	*b*	Δ*b*
(HCN)_2_	2.15	180	180	1.084	0.007
(HSiN)_2_	2.21	180	163	1.497	0.007
(HGeN)_2_	2.09	180	180	1.532	0.007
(HSnN)_2_	1.91	180	133	1.703	0.034
(HNC)_2_	2.03	180	180	1.023	0.017
(HNSi)_2_	2.78	180	180	1.012	0.001
(HNGe)_2_	2.63	180	180	1.015	0.001
(HNSn)_2_	2.84	180	180	1.019	0.000
(HNPb)_2_	2.84	180	180	1.023	−0.001

a Distances are given in *A* and angles in degrees.

**Fig. 3:**
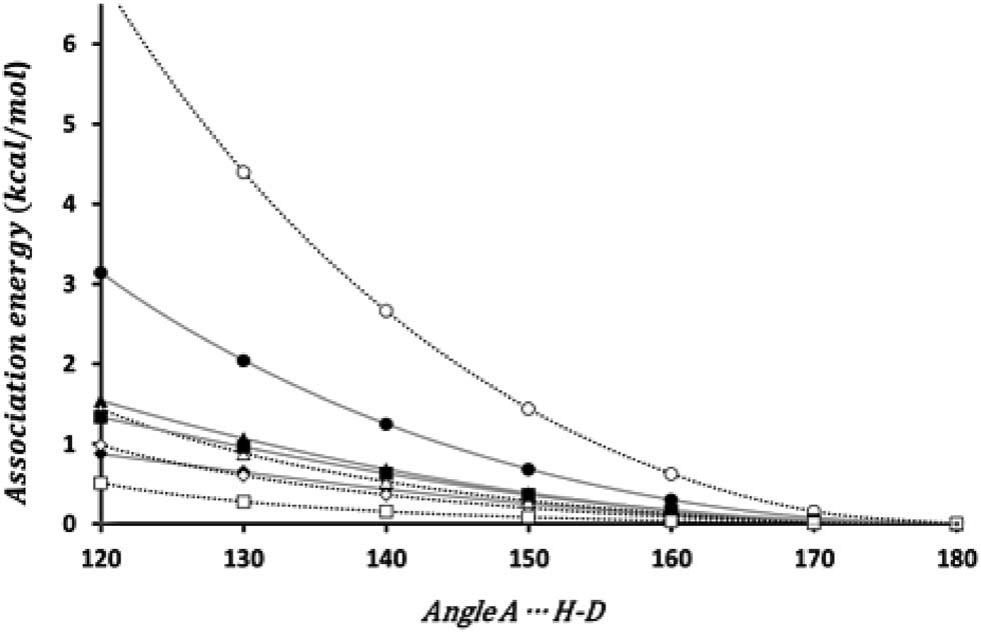
Angular dependence of the association energy. Black circle (HCN)_2_; black diamond (HSiN)_2_; black triangle (HGeN)_2_; black square (HSnN)_2_; white circle (HNC)_2_; white diamond (HNSi)_2_; white triangle (HNGe)_2_; white square (HNSn)_2_.

The bond directionality, or the variation of the association energy with respect to the angle *ϕ* is a characteristic of HBs. We have estimated the change in the association energy upon changing the *ϕ* angle but keeping the *θ* angle at the value in the equilibrium geometry. As can be seen in Fig. 3 the association energy of all the investigated dimers presents angular dependence, although for some systems the energetic change barely reach 0.5 kcal mol^−1^ upon changing *ϕ* from 180° to 120°, but for the set of energetically favorable dimers the change is of 1 kcal mol^−1^, at least. Thus all these interactions fulfill one of the distinctive characteristics of HBs.

Another two important characteristics of HB bonded systems is the change in the stretching frequency of the *b* bond upon the dimer formation, as well as the non-linear addition of the monomer dipole moments upon dimer formation. In Table 3 are listed the *b* bond stretching frequencies for the isolated monomers (*ν*), calculated at the MP2/aug-cc-pVDZ level of theory and the change in *ν* upon the dimer formation (Δ*ν*). In all the cases *ν* change upon the dimer formation and it shifts to the red; *i.e.* Δ*ν* is negative. However for the energetically favorable dimers the shift is considerably larger, at least double, than for the rest of the dimers. The change in the dipole moment (Δ*μ*) upon the dimer formation, measured as Δ*μ* = *μ*_d_ − 2*μ* where *μ*_d_ is the dipole moment of the dimer and *μ* is the dipole moment of the monomer, is listed in Table 3 along with the *μ* values. The largest values of Δ*μ* are also found for the energetically favorable dimers, in these cases the dipole moment changes around 1 debye or more upon dimer formation, which represents an increment of *μ* between 27% and 41%. For the rest of the dimers *μ* changes only 18%, at most, upon the dimer formation.

**Table 3:** Proton stretching frequencies (*ν*, in cm^−1^), the change in upon de dimer formation (Δ*ν*, in cm^−1^), and changes in the dipole moment (Δ*μ*, in debye) upon the dimer formation

Monomer	*ν*	Δ*ν*	*μ*	Δ*μ*
HCN	3462.87	−94.62	3.33	0.92
HSiN	2225.52	−53.24	5.96	1.85
HGeN	2169.86	−80.50	5.89	2.30
HSnN	2087.20	−371.86	6.29	2.01
HNC	3801.12	−321.53	2.78	1.15
HNSi	3715.16	−21.78	0.99	−0.11
HNGe	3669.31	−26.65	1.47	−0.05
HNSn	3605.98	−14.58	2.88	0.33
HNPb	3556.50	−8.55	3.83	0.69

The presence of a bond critical point (BCP), as defined by QTAIM, between the proton and the acceptor atom, together with specific values of the electronic charge density and its Laplacian at the BCP, *ρ*_BCP_ and Δ*ρ*_BCP_ respectively, are considered to be characteristics of the an HB formation. According to Koch and Popelier *ρ*_BCP_ and Δ*ρ*_BCP_ should be in the range of [0.002, 0.035] a.u. and [0.0124, 0.139] a.u. respectively.^18^ Analyzing the electronic charge density obtained at the CCSD(T)/aug-cc-pVQZ level of theory we have found BCPs between the proton and the acceptor atom in all the investigated dimers, and according to the values of *ρ*_BCP and Δ*ρ*_BCP__, listed in Table 4, all these systems are forming HBs.

**Table 4:** Values of the density of at the intermolecular BCP in a.u. for HXN and HNX systems

Dimer	*ρ*_IBCP_	Δ*ρ*_IBCP_
(HCN)_2_	0.017	0.061
(HSiN)_2_	0.017	0.052
(HGeN)_2_	0.021	0.061
(HSnN)_2_	0.026	0.069
(HNC)_2_	0.025	0.065
(HNSi)_2_	0.009	0.022
(HNGe)_2_	0.012	0.029
(HNSn)_2_	0.009	0.023
(HNPb)_2_	0.009	0.025

**Fig. 4:**
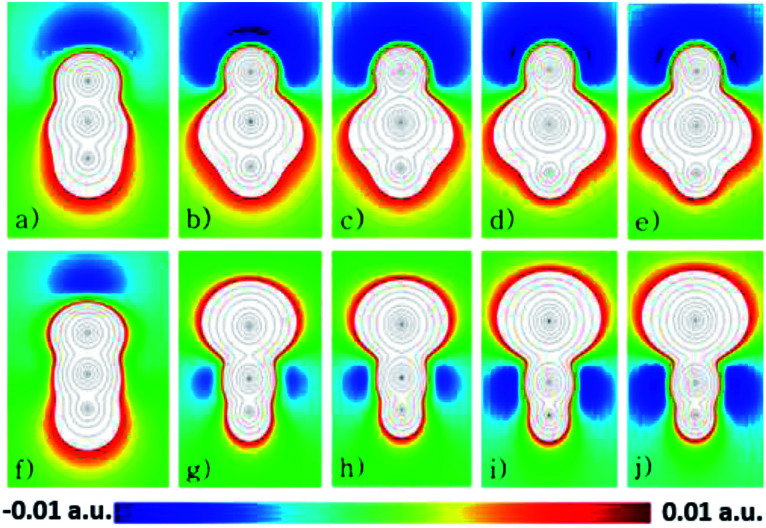
Representation of the EP map, (a) HCN, (b) HSiN, (c) HGeN, (d) HSnN, (e) HPbN, (f) HNC, (g) HNSi, (h) HNGe, (i) HNSn and (j) HNPb. Red dots are the minima, and the blue the maxima, while the light blue is the second and third maxima respectively and the orange and green are the second minima.

As an attempt to understand why the HXN dimers interact strongly than the HNX ones, except for the HNC dimer, it is analyzed the electrostatic potential of the monomers, calculated at the CCSD(T)/aug-cc-pVQZ level of theory. In Fig. 4 are shown contour diagrams of the electrostatic potential for all the investigated systems, including the HPbN one. The contours of the electrostatic potential lay along a plane that passes through all the atoms and its covalent bonds. In these plots it is clearly seen that the negative part of the electrostatic potential is around the N atom in all the cases except in the HNC molecule. Therefore in the HXN and in the HNC molecules the negative part of the potential lay in the acceptor atom, in the other systems the electrostatic potential is positive around the acceptor atom. These results and the interaction energies clearly suggest that the HXN dimers and the HNC one are hydrogen bonded while the rest do not. According to the electrostatic potential the HPbN⋯HPbN dimer should be stable, however that system transforms into the HNPb⋯HNPb as it is mentioned above. Likely the latter behavior results from the position of the minimum of the electrostatic potential. The distance from that minimum to the acceptor atom (*d*_mep_) in the energetically favorable dimers is listed in Table 5. The *d*_mep_ values can be grouped in two sets, the *d*_mep_ large values that are around 1.9 and 2 Å and the *d*_mep_ small values which are around 1.4 and 1.5 Å. The HSnN and HNC systems are among these with small *d*_mep_ values. According to the data presented in Table 2, these systems present the larger elongation of the *b* bond upon the dimer formation, moreover these systems also present the largest changes in *ν* (see Table 3). The HPbN is the system with the smallest *d*_mep_ value, hence suggesting that in the process of the dimer formation, the proton attracted by the minimum of the electrostatic potential gets so close to the acceptor atom (N) that covalently bond each other. In the systems with *d*_mep_ large values the changes in *ν* and *b* are three times smaller, at least upon the dimer formation than those in the systems with *d*_mep_ small values.

**Table 5:** Distance, in Å, between the acceptor atom and the minimum of the electrostatic potential

Monomer	*d*_mep_
HCN	2.00
HSiN	1.90
HGeN	1.90
HSnN	1.41
HPbN	1.36
HNC	1.50

The energetics and the analysis of the electrostatic potential presented above suggest that the systems HNC⋯HNC, HSiN⋯HSiN, HGeN⋯HGeN, HCN⋯HCN and HSnN⋯HSnN are hydrogen bonded. Moreover we present evidence of the HB formation in these systems analyzing the change in *ν* and *b* upon the dimer formation, the bond directionality, the presence of BCPs between the proton and the acceptor atom and values of *ρ*BCP and Δ*ρ*BCP within the expected ranges. Considering the electronegativity values reported in ref. 19, the latter thus illustrates the formation HBs between systems in which the donor atom is less electronegative than the H atom, as in the HSiN⋯HSiN, HGeN⋯HGeN and the HSnN⋯HSnN dimers, situation that it is not contemplated in the accepted criteria for determining the HB formation. Therefore these HBs can be considered as a new group of non-conventional ones. Also our results show that if the acceptor atom is less electronegative than the H atom, the HB seems not to be formed.

## 4. Conclusions

In conclusion in this work it is shown that the systems HNC, HSiN, HGeN, HSnN and HNC are form HBs with itself, while the systems HNSi, HNGe, HNSn and HNPb do not. The latter thus show that systems in which the donor atom is less electronegative than the H atom, like in HSiN, HGeN and HSnN, are able to form HBs, which broaden our understanding of the hydrogen-bonding phenomenon.

## Conflicts of interest

There are no conflicts to declare.

## Supplementary Material

RA-009-C9RA00856J-s001
