# Supplementary material for: Hydrogen bonding capabilities of group 14 homologues of HCN and HNC
Source: RSC Adv. 2019 Feb 18;9(11):5937–41. doi: 10.1039/c9ra00856j (PMC9060874; doi:10.1039/c9ra00856j)
Supplement: RA-009-C9RA00856J-s001 [file RA-009-C9RA00856J-s001.pdf]

Electronic Supplementary Material (ESI) for RSC Advances.
This journal is © The Royal Society of Chemistry 2019

Hydrogen bonding capabilities of group 14 homologues of HCN and HNC

Joanatan M. Bautista-Renedo, Horacio Reyes-Pérez, Erick Cuevas-Yáñez, Carlos Barrera-Díaz, Nelly González-Rivas and Joel Ireta

email: nelymagr@gmail.com

Energies and coordinates of the calculated dimers

$(HCN)_2$ dimmer

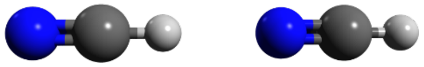

```
E(BSSE Corrected  MP2/aug-cc-pVDZ//CCSD(T)/aug-cc-pVQZ)= -186.6117560 Ha
H 0.0000000000 0.0000000000 -3.7949516300
C 0.0000000000 0.0000000000 -2.7180143800
N 0.0000000000 0.0000000000 -1.5386830800
H 0.0000000000 0.0000000000  0.6078420000
C 0.0000000000 0.0000000000  1.6916814900
N 0.0000000000 0.0000000000  2.8736983500
```

$(HSiN)_2$ dimmer

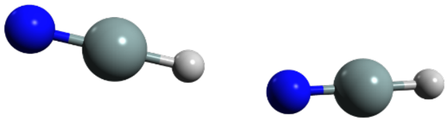

```
E(BSSE Corrected  MP2/aug-cc-pVDZ//CCSD(T)/aug-cc-pVQZ)= -688.4614417 Ha
Si -0.3510000000 -3.0730000000 0.0000000000
Si  0.2940000000  2.1770000000 0.0000000000
N  -0.4850000000 -1.4430000000 0.0000000000
N   0.6290000000  3.7850000000 0.0000000000
H  -0.2200000000 -4.5570000000 0.0000000000
H   0.0000000000  0.7090000000 0.0000000000
```

$(HGeN)_2$ dimmer

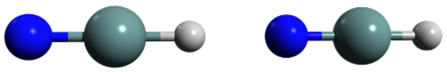

```
E(BSSE Corrected  MP2/aug-cc-pVDZ//CCSD(T)/aug-cc-pVQZ)= -4261.662058 Ha
H  0.0000000000 0.0000000000  4.4462977100
Ge 0.0000000000 0.0000000000  2.9214801200
N  0.0000000000 0.0000000000  1.2235170700
```

H	0.0000000000	0.0000000000	-0.8692058500
Ge	0.0000000000	0.0000000000	-2.4016481400
N	0.0000000000	0.0000000000	-4.1109049800

$(HSnN)_2$ dimmer

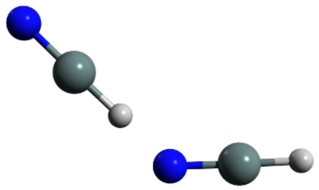

E(BSSE Corrected MP2/aug-cc-pVDZ//CCSD(T)/aug-cc-pVQZ)= -537.4864554 Ha

Sn	1.1670000000	2.0560000000	0.0000000000
Sn	-1.3010000000	-2.3960000000	0.0000000000
N	2.4900000000	3.4180000000	0.0000000000
N	-1.3590000000	-0.5300000000	0.0000000000
H	0.0000000000	0.8150000000	0.0000000000
H	-1.2150000000	-4.0630000000	0.0000000000

$(HNC)_2$ dimmer

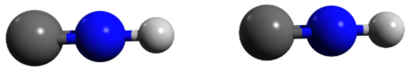

E(BSSE Corrected MP2/aug-cc-pVDZ//CCSD(T)/aug-cc-pVQZ)= -186.5695365 Ha

H	0.0000000000	0.0000000000	3.5618994900
N	0.0000000000	0.0000000000	2.5557680100
C	0.0000000000	0.0000000000	1.3698327800
H	0.0000000000	0.0000000000	-0.6594665500
N	0.0000000000	0.0000000000	-1.6823277500
C	0.0000000000	0.0000000000	-2.8725852400

$(HNSi)_2$ dimmer

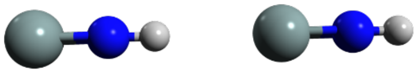

E(BSSE Corrected MP2/aug-cc-pVDZ//CCSD(T)/aug-cc-pVQZ)= -688.6733033 Ha

H	0.0000000000	0.0000000000	-4.6679536400
N	0.0000000000	0.0000000000	-3.6572584400
Si	0.0000000000	0.0000000000	-2.0691857000
H	0.0000000000	0.0000000000	0.7142637600
N	0.0000000000	0.0000000000	1.7260077000
Si	0.0000000000	0.0000000000	3.3172175000

$(HNGe)_2$ dimmer

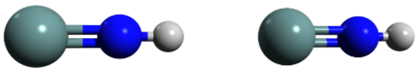

E(BSSE Corrected MP2/aug-cc-pVDZ//CCSD(T)/aug-cc-pVQZ)= -4261.8605290 Ha

H	0.0000000000	0.0000000000	-4.9943781800
N	0.0000000000	0.0000000000	-3.9804115500

```
Ge 0.0000000000 0.0000000000 -2.3017368100
H  0.0000000000 0.0000000000  0.3265298500
N  0.0000000000 0.0000000000  1.3417870800
Ge 0.0000000000 0.0000000000  3.0248061800
```

$(HNSn)_2$ dimmer

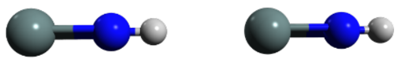

```
E(BSSE Corrected  MP2/aug-cc-pVDZ//CCSD(T)/aug-cc-pVQZ)= -537.6974426 Ha
H  0.0000000000 0.0000000000 -5.5081804700
N  0.0000000000 0.0000000000 -4.4891063400
Sn 0.0000000000 0.0000000000 -2.5971742100
H  0.0000000000 0.0000000000  0.2392024100
N  0.0000000000 0.0000000000  1.2584780000
Sn 0.0000000000 0.0000000000  3.1548417400
```

$(HNPb)_2$ dimmer

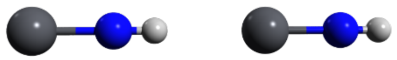

```
E(BSSE Corrected  MP2/aug-cc-pVDZ//CCSD(T)/aug-cc-pVQZ)= -494.8606690 Ha
H  0.0000000000 0.0000000000 -5.7554648500
N  0.0000000000 0.0000000000 -4.7316109000
Pb 0.0000000000 0.0000000000 -2.7409907800
H  0.0000000000 0.0000000000  0.0991947000
N  0.0000000000 0.0000000000  1.1222635600
Pb 0.0000000000 0.0000000000  3.1180847000
```
